# The effect of ghrelin on antioxidant status in the rat’s model of Alzheimer’s disease induced by amyloid-beta

**DOI:** 10.37796/2211-8039.1341

**Published:** 2022-12-01

**Authors:** Fatemeh Sarlaki, Zahra Shahsavari, Fatemeh Goshadrou, Faezeh Naseri, Mohammad Keimasi, Majid Sirati-Sabet

**Affiliations:** a Department of Clinical Biochemistry, School of Medicine, Shahid Beheshti University of Medical Sciences, Tehran, Iran; b Department of Physiology, School of Allied Medical Sciences, Shahid Beheshti University of Medical Sciences, Tehran, Iran; c Department of Pharmacology and Toxicology and Isfahan Pharmaceutical Sciences Research Center, School of Pharmacy and Pharmaceutical Sciences, Isfahan University of Medical Sciences, Isfahan, Iran

**Keywords:** Alzheimer’s disease, Ghrelin, Lipid peroxidation, Antioxidant capacity, Catalase

## Abstract

Alzheimer’s disease (AD) is a neurodegenerative disorder associated with amyloid-beta (Aβ) plaque formation and oxidative stress in the brain. Ghrelin has been proven to exert antioxidant activity and neuroprotection in different neurological diseases. This study is going on to examine the effect of ghrelin on antioxidant status in the rat’s model of AD induced by Aβ. Cognitive impairment was induced by intra-hippocampal administration of Aβ (10 μg) in Wistar rats and ghrelin (80 μg/kg) was administrated intraperitoneal for ten consecutive days. Behavior was assessed with Morris water maze and passive avoidance tests. Malondialdehyde (MDA) level as a marker of lipid peroxidation was assessed using the thiobarbituric acid. Catalase activity was assayed by the decomposition of H_2_O_2_. Antioxidant capacity was determined using the FRAP method. Treatment with ghrelin decreased the hippocampus and serum MDA levels in wild-type rodents and prevented an increase in hippocampal and serum MDA levels in animals receiving Aβ. There was no significant change in the serum catalase activity between the studied groups. Hippocampus catalase activity was reduced in the Aβ group and treatment with ghrelin increased it. The antioxidant capacity of the hippocampus and serum increased in the ghrelin-receiving control group. The hippocampus antioxidant capacity level decreased in the Aβ group, and treatment with ghrelin increased it, but there were no significant changes in the serum antioxidant capacity of animals receiving Aβ. These results provide evidence that the administration of ghrelin has antioxidant properties and protects against hippocampal lipid peroxidation in a rat model of AD.

## 1. Introduction

Alzheimer’s disease (AD) is the most common form of dementia, in which amyloid-beta (Aβ) plaques are found in the brain. One of the complications of AD is the deterioration of mental functions in the individual. Various cognitive abilities, such as memory, language, argument, and decision-making, are impaired with dementia [1]. Gradual memory impairment, one of the early signs of AD, has been attributed to the deposition of Aβ plaques in the brain, especially in the hippocampus. Two proteases, beta, and gamma-secretase produce Aβ peptide by the amyloid precursor protein (APP) breakdown [2]. Increased production or defect in the clearance of Aβ causes the aggregation of toxic amounts of Aβ, which results in the formation of plaques containing Aβ in the brain which, in turn, causes nerve damage and dementia [3]. Amyloid plaques accumulate mainly in the isocortex in AD [4]. In the Aβ plaques cascade hypothesis, the accumulation of Aβ, especially Aβ 1–42 in the brain, creates a cascade of events that lead to neuronal dysfunction and destruction [5,6]. Some studies have shown that oxidative stress may be one of the mechanisms of Aβ to induce neuronal damage [7,8].

Overproduction of reactive species, including free radicals, and a weakening of antioxidant defense systems are observed during oxidative stress [9]. Increasing reactive oxygen species (ROS) can play a vital role in the pathogenesis of AD [10]. These compounds cause damage to biological molecules [11]. Some compounds and enzymes are involved in the removal of ROS [12] such as catalase (CAT), superoxide dismutase (SOD), and glutathione peroxidase (GPX) [13]. The activity of enzymes involved in the antioxidant system has decreased in AD, leading to oxidative stress [14].

ROS can be created by damaged mitochondria or produced by the innate immune in response to inflammatory stimuli. Aβ and hyperphosphorylated tau protein can play an essential role in mitochondrial dysfunction before the onset of amyloid plaques. Aβ can trigger mitochondrial dysfunction by interrupting the activity of complex III and complex IV, and inhibiting directly the activity of the pyruvate dehydrogenase and α-ketoglutarate dehydrogenase enzymes. Aβ also disrupts mitochondrial calcium homeostasis and mitochondrial permeability transition pore [15]. Some evidence has shown that mitochondrial dysfunction leads to increased ROS production leading to the early stages of AD [9]. Astrocytes and microglia are other sources of ROS and RNS in the brain that can produce free radicals when activated [15]. Oxidative stress occurs in the early stages of AD and is associated with the presence of Aβ as the cause of AD pathogenesis [16]. Aβ can be the source of ROS and the initiator of free radical damage to biomolecules in the brain, including fat peroxidation. In normal conditions, antioxidant defense systems eliminate overproduced ROS [10]. Excess Aβ is associated with increased oxidation of proteins, fats, and nucleic acids in the hippocampus of Alzheimer’s patients [16]. Oxidative changes in RNA and DNA in the nucleus and mitochondria, high levels of oxidized proteins, free carbonyls, glycosylated products, peroxidation of fats, and formation of aldehydes and ketones are markers of oxidative stress. Some oxidative stress markers increase in people’s brain tissue in the early or clinical stages of AD [17]. Evidence of low antioxidant capacity in the brain, CSF, and blood of Alzheimer’s patients has also been reported [18,19].

Ghrelin is a peptide hormone consisting of 28 amino acids secreted from gastric enteroendocrine cells during starvation. Ghrelin exists as acyl ghrelin and non-acylated (inactive) ghrelin [20]. Acyl ghrelin makes up only 10% of the circulating ghrelin. In addition to the stomach, this hormone is secreted from some other tissues in the body. It has several biological effects, including appetite regulation, glucose metabolism, and energy homeostasis in the hypothalamus [21]. Also, ghrelin and its receptor (GHSR-1) are abundant in brain areas involved in memory and learning and can affect neuronal activity and neurotransmitter release [22,23]. The protective effect of ghrelin on neurons and its association with AD has been identified [24]. This hormone has been reported to affect oxidative stress. Ghrelin prevents a decrease in antioxidant enzyme activity in rat plasma [25]. This hormone reduces the amount of fat peroxidation in the rat plasma [25] and increases the antioxidant defense in the liver of rats [12]. It also has an antioxidant effect on the ovary and testis of rats [26,27].

Considering the antioxidant effect of ghrelin in various studies and the protective role of this hormone on neurons and its relationship with AD, ghrelin’s effect on the inhibition of oxidative stress in Aβ-induced AD rats was investigated in the present study. First, after a histopathological study, we examined the effects of ghrelin on memory and learning. In the next step, we assessed the effect of ghrelin treatment on the malondialdehyde (MDA) level as a marker of lipid peroxidation, catalase activity, and antioxidant capacity in the extracted hippocampus and serum of Aβ-induced AD rats.

## 2. Method

### 2.1. Animals

Thirty adult male Wistar rats (250 ± 20 g) were purchased from Pasteur Institute (Tehran, Iran). They were housed in cages of three in a temperature-controlled holding room (23 ± 1 °C), with humidity ~60%, and on a 12/12 h light/dark cycle (lights on at 8:00 AM). Standard laboratory procedures were followed. Food ad libitum and water were available. All experiments were performed following the guidelines for the care and use of laboratory animals at Shahid Beheshti University of Medical Sciences.

### 2.2. Drugs and agents

All chemicals were purchased from Merck unless otherwise specified. Aβ peptide 1–42 (Sigma–Aldrich, Munich, Germany) was dissolved in sterile PBS at a 2 mg/ml concentration and was incubated at room temperature for 36 h [28]. Acylated ghrelin of rat (GL Biochem Ltd, Shanghai, China) prepared in sterile normal saline at 1 mg/ml concentration and kept at −20 °C until use. Ketamine 10% and xylazine 2% were supplied by Alfasan (Woerden, Holland), and persocaine-E was purchased from Darou Pakhsh (Tehran, Iran).

### 2.3. Experimental design

In this pilot study, the rats were randomly sorted into five different groups, six rats in each. Each experiment was conducted according to the following schedule:

-The control group: The animals were intact and did not receive any treatment.
-The sham group: Received 5 μl PBS in the hippocampus and, after one day, received daily intraperitoneal (i.p.) injections of 20 μl of saline for ten consecutive days.
-The Ghrelin group: Received 5 μl PBS in the hippocampus and, after one day, received a daily i.p injection of 80 μg/kg of ghrelin for ten consecutive days.
-The Aβ group: Received a 10 μg (2 mg/ml) injection of Aβ 1–42 in the hippocampus and, after one day, were i.p injected with a daily dose of 20 μl of saline for ten consecutive days.
-The Ghrelin-treated (Aβ + Ghrelin) group: Received a 10 μg (2 mg/ml) injection of Aβ 1–42 in the hippocampus and, after one day, were i.p. injected with 80 μg/kg of ghrelin every day for ten consecutive days.

**Fig. 1:**
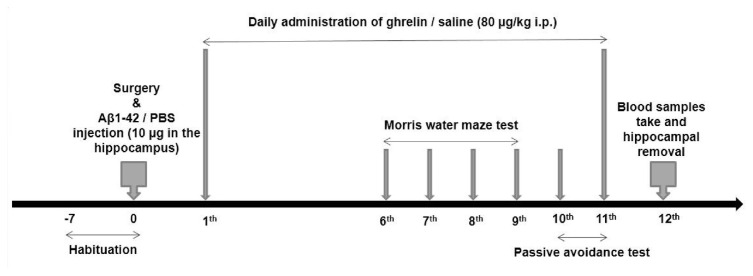
Animal study design.

After performing behavioral tests (twelve days after surgery), blood samples were taken from the retro-orbital plexus, and serum was prepared. At the end of the experiment, rats were anesthetized with a ketamine/xylazine mixture and were sacrificed. Immediately, the hippocampus was excised and frozen in liquid nitrogen. The RIPA lysis buffer containing a protease inhibitor cocktail was used for homogenizing samples. The homogenates were centrifuged at 12000g at 4 °C [29]. The supernatant and serum were used to determine the catalase activity, the antioxidant capacity, and the malondialdehyde (MDA) level (Fig. 1).

### 2.4. Stereotaxic surgery

Before surgery, each rat was anesthetized with an i.p. injection of ketamine (85 mg/kg) and xylazine (15 mg/kg). Adequate persocaine-E was injected subcutaneously to prevent bleeding and induce local anesthesia.

### 2.5. Micro-injection procedure

**Fig. 2:**
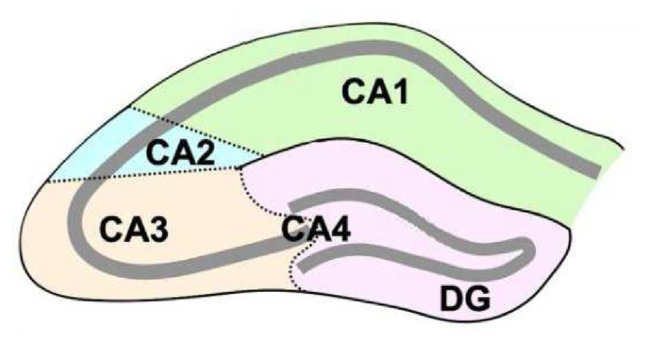
Diagram of the major divisions of the hippocampus. CA, Cornu Ammonis; DG, dentate gyrus [31].

Each rat was mounted on a stereotaxic apparatus (Stoelting, USA), and aggregated Aβ 1–42 or PBS was injected bilaterally into the Cornu Ammonis 3 (CA3) area of the hippocampus using a 5 μl Hamilton syringe (Fig. 2). The injection site was identified on the skull as follows: AP: −3.3 mm from the bregma; ML: ±2.6 mm from the midline; DV: 3.7 mm from the skull surface based on Paxinos and Watson’s Atlas [30].

### 2.6. Histological evaluation

Histological studies were performed on the brain tissue of some rats after surgery and Aβ injection. Rats were sacrificed in CO_2_ chambers, and the brains were removed and stabilized. Then, dehydration, clarification, and molding steps were performed, and 5 μm thick sections were prepared with a microtome. Nissl staining was used to stain nissl bodies in the cytoplasm of neurons. For cell count, identical sections (three rats in each group) were used, and the number of dark cells/mm^2^ was determined.

### 2.7. Behavioral studies

#### 2.7.1. Morris water maze test

The Morris water maze (MWM) test began six days after surgery. The water maze apparatus consisted of a black metal tank and habituation was done one day before the training began. The daily interval between tests was 30 s and the rat was allowed to stay on the platform for 20 s before removal from the tank. The training phase lasted for three days. On the probe day, the time spent in the target quadrant was recorded [32].

#### 2.7.2. Passive avoidance test (PAT)

The passive avoidance behavior was done ten days after surgery and was performed on two consecutive days. The apparatus that was used to evaluate the passive avoidance memory performance had two equal-sized light and dark compartments. The dark compartment was connected to a shock source. Before the training trials, animals were habituated to the apparatus by placing them in the lightroom. After 5 s, the door was elevated. When the rat entered the dark compartment with all four feet, the door was closed, and the rat remained there for 20 s. After that, the animal was put into a temporary cage. After 30 min, the rat was again confined in the white compartment for 5 s. The door was opened to let the animal enter the dark chamber, and after the entrance, the door was shut, but this time animals received a foot shock (1.2 mA, 50 Hz, and 1.5 s). After 20 s, the rat was returned to the temporary cage. After 2 min, the same testing process was reiterated. The rats were again placed in the light chamber to see whether they stayed there for 2 min or not. It was considered successful learning when the animals did not enter the dark compartment during that period; otherwise, they would receive the same electric shock again until they refrain from entering the dark box. The memory retrieval test was performed to assess the long-term memory of the animal 24 h after learning. At this stage, the delay time of the animal for the first entry into the dark chamber was assessed and the step-through latency (STL) was recorded for 10 min [32].

### 2.8. Measurement of lipid peroxidation

Lipid peroxidation was evaluated by measuring the amount of malondialdehyde (MDA) by the modified methods of Ohkawa based on TBA reactivity [33]. The standard curve is prepared with 1,1,3,3-tetraethoxypropane (Sigma–Aldrich, Munich, Germany) solution. Briefly, serum or hippocampal lysate (0.1 ml), SDS, and butylated hydroxytoluene were mixed and then TBA in acetate buffer (pH 3.0) was added. The tubes were incubated at 95 °C in a water bath for 60 min and after centrifugation, the supernatant was taken. After this, the solution’s adsorption was read at 532 and 572 nm, and the adsorption difference (A_532_ – A_572_) was used in the calculations.

### 2.9. Catalase activity assay

Catalase activity was assayed by the decomposition of H_2_O_2_ as catalase substrate. Briefly, 20 μl of the sample was incubated with 100 μl of hydrogen peroxide (65 μmol/ml in 60 mmol/L sodium-potassium phosphate buffer pH 7) for 1 min at 37 °C. After adding 100 μl of 4% ammonium molybdate the yellow complex was measured at 405 nm. One catalase activity unit was determined as the amount of enzyme required to decompose one μmol hydrogen peroxide/min at 37 °C [34].

### 2.10. Measurement of antioxidant capacity

Antioxidant capacity was measured by the ferric reducing antioxidant power (FRAP) assay. In this method, the reduction of ferric ion (Fe^3+^) to the ferrous ion (Fe^2+^) that forms a blue complex with TPTZ (2,4,6-tri[2-pyridyl]-s-triazine) measures in an acidic medium at 593 nm. The standard curve was drawn using a ferrous sulfate solution. The FRAP solution contains acetate buffer (pH 3.6), TPTZ, and ferric chloride. Briefly, 10 μl serum was added to 300 μl FRAP solution at 37 °C, and its absorption was read in front of the blank after 5 min at 595 nm [35].

### 2.11. Measurement of protein concentration

The protein concentration was determined according to the Bradford method [36]. The standard curve was prepared using bovine serum albumin.

### 2.12. Statistical analysis

Data analysis was done through GraphPad Prism software, version 8.0. The results are expressed as the mean ± SD. The t-test and one-way analysis of variance (ANOVA) followed by Tukey’s test were used for data analysis. The statistical significance was achieved when *p* < 0.05.

## 3. Results

### 3.1. Histological studies

**Fig. 3:**
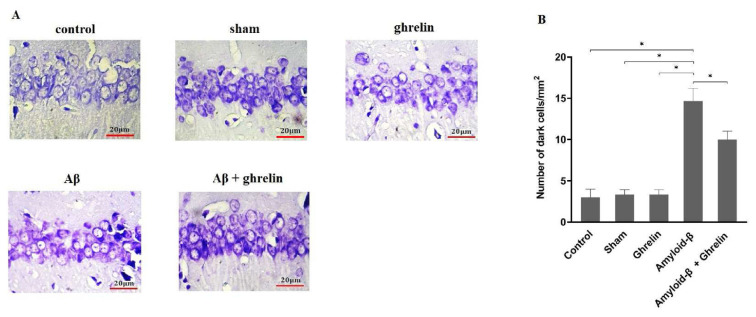
Histological studies. (A) Nissl staining of CA1 region of rat hippocampus in the control, sham, ghrelin, Aβ, and Aβ + ghrelin groups, and (B) Number of dark cells/mm^2^ in CA1 region of rat hippocampus in the control, sham, ghrelin, Aβ, and Aβ + ghrelin groups (n = 3). The data are shown as means ± SD. *p < 0.05.

Histological examination with nissl staining in the control, sham, ghrelin, and ghrelin treatment groups shows pyramidal neurons with clear cytoplasm in the CA1 region of the hippocampus. The size of pyramidal neurons was smaller, the nuclei were wrinkled, the cytoplasmic range of the cell was not well defined in some cases and overall cell density in the Aβ group was lower than other groups in the nissl staining of the CA1 region of the hippocampus (Fig. 3A).

There was a significant difference (p < 0.05) in the number of dark cells/mm^2^ in the CA1 region of the hippocampus between the control, sham, and ghrelin groups with the Aβ group. Ghrelin treatment (Aβ+ghrelin group) significantly (p<0.05) decreased the number of dark cells/mm^2^ in the CA1 region of the hippocampus compared to the Aβ group (Fig. 3B).

### 3.2. The effects of ghrelin in behavioral studies

**Fig. 4:**
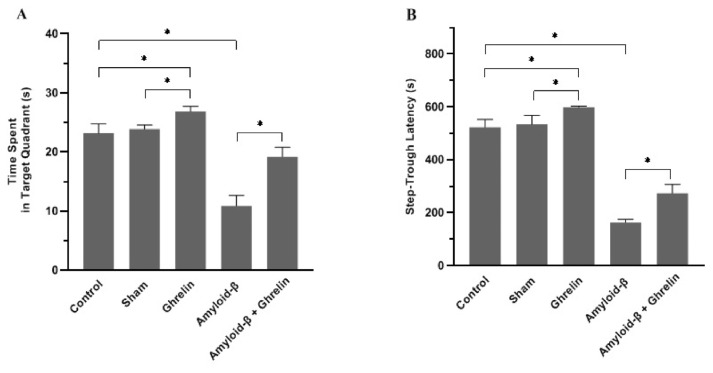
Behavioral studies. (A) Time spent in the target quadrant, (B) Step-through latency (STL) in the control, sham, ghrelin, Aβ, and Aβ + ghrelin groups (n = 6). The data are shown as means ± SD. *p < 0.05.

The Morris water maze (MWM) test is used to test the animal’s spatial memory. There was no significant difference in the target quadrant’s time in the control and sham groups during the probe test. The time spent in the target quadrant was significantly increased (p < 0.05) in rats in the ghrelin group compared to the control and sham groups. The time spent in the target quadrant was reduced significantly (p < 0.05) in rats receiving Aβ, and ghrelin treatment (Aβ + ghrelin group) significantly (p < 0.05) increased it compared to the Aβ group (Fig. 4A).

As shown in Fig. 4B, there was no significant difference in step-through latency (STL) in the control and sham groups on the passive avoidance test. Ghrelin caused a significant (p < 0.05) increase in STL in rats compared to control and sham groups. STL was reduced significantly (p < 0.05) in the Aβ group compared to the other groups. Treatment with ghrelin (Aβ + ghrelin group) significantly increased STL (p < 0.05) with respect to the Aβ group.

### 3.3. The effects of ghrelin on lipid peroxidation

**Fig. 5:**
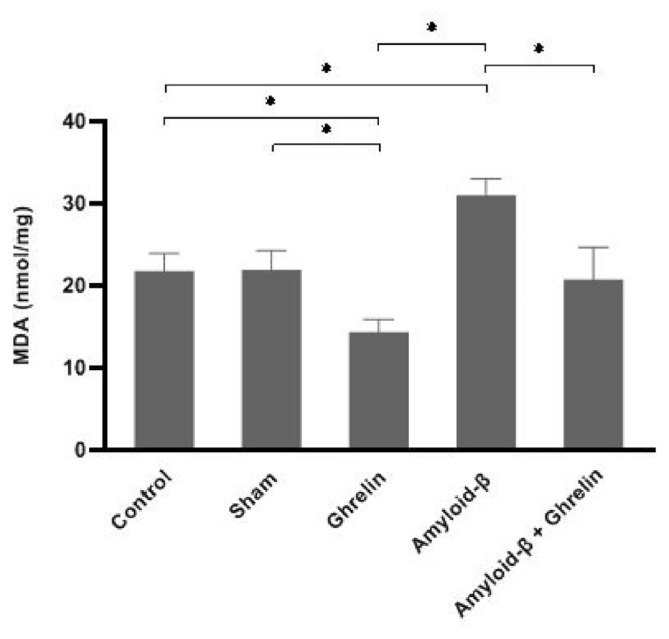
The MDA level (nmol/mg protein) in the hippocampus of rats in the control, sham, ghrelin, Aβ, and Aβ + ghrelin groups (n = 6). The data are shown as means ± SD. *p < 0.05.

The hippocampus and serum MDA levels in rats in the ghrelin group were significantly reduced (p < 0.05) compared to the control, sham, and Aβ groups. The amount of MDA in the hippocampus and serum of rats in the Aβ group was significantly increased (p < 0.05) compared to the control, sham, and ghrelin groups. Administration of ghrelin (Aβ + ghrelin group) significantly (p < 0.05) decreased the hippocampus and serum MDA level compared to the Aβ group (Fig. 5 and Table 1).

### 3.4. The effects of ghrelin on catalase activity

**Fig. 6:**
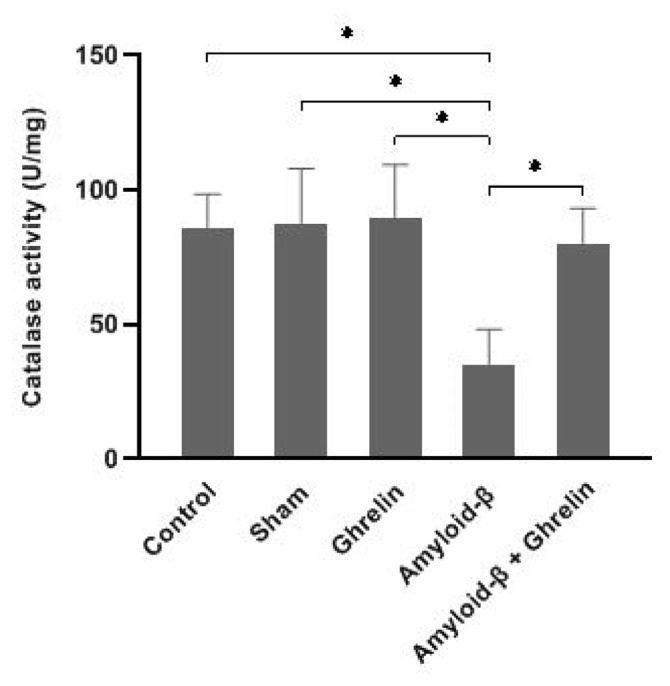
The catalase activity (U/mg protein) in the hippocampus of rats in the control, sham, ghrelin, Aβ, and Aβ + ghrelin groups (n = 6). The data are shown as means ± SD. *p < 0.05.

There was no significant change in serum catalase activity between the studied groups (Table 1). The hippocampus catalase activity was reduced significantly (p < 0.05) in rats in the Aβ group compared to the control, sham, and ghrelin groups. Treatment with ghrelin (Aβ + ghrelin group) significantly increased hippocampus catalase activity (p < 0.05) compared to the Aβ group (Fig. 6).

### 3.5. The effects of ghrelin on antioxidant capacity

**Fig. 7:**
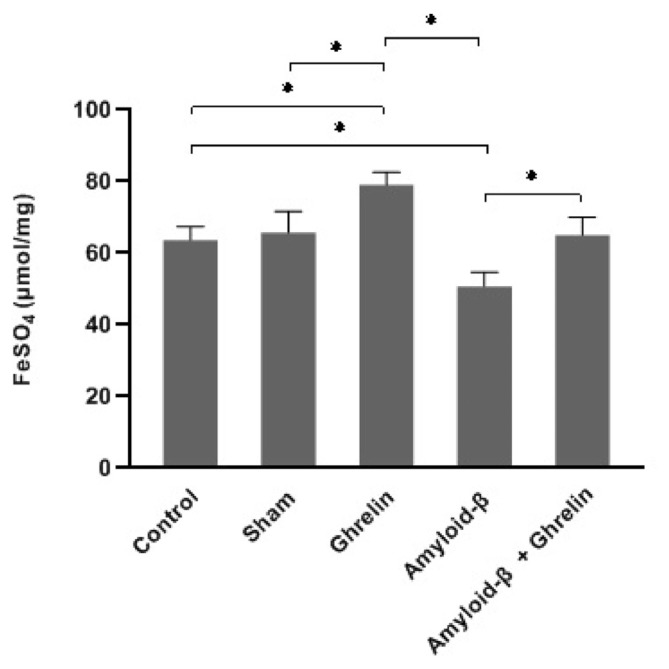
The antioxidant capacity (μmol/mg protein) in the rats’ hippocampus in the control, sham, ghrelin, Aβ, and Aβ + ghrelin groups (n = 6). The data are shown as means ± SD. *p < 0.05.

Ghrelin caused a significant (p < 0.05) increase in antioxidant capacity level in the hippocampus and serum of rats compared to the control and sham groups, while Aβ did not result in any change in this parameter in the serum. The hippocampus antioxidant capacity level significantly decreased (p < 0.05) in the Aβ group compared to the control, sham, and ghrelin groups, and treatment with ghrelin (Aβ + ghrelin group) significantly increased (p < 0.05) this parameter (Fig. 7 and Table 1).

## 4. Discussion

AD is a progressive and chronic disease of the nervous system, and its clinical features include memory loss and personality changes [37]. In this experimental study, Aβ 1–42 was used to create a similar Alzheimer’s condition in the rat model and ghrelin reduces oxidative stress due to its antioxidant properties and inhibiting fat peroxidation in the brain. The majority of pyramidal neurons in the hippocampus are densely packed into a single layer divided into CA1 through CA4 regions. CA1 neurons are more sensitive to oxidative stress [38]. In the cytoplasm of neurons, nissl bodies are stained (blue-purple) by the nissl staining. This staining can typically be used to detect necrosis in brain tissue [39]. In the present study, histological studies on rats receiving Aβ indicate changes in CA1 hippocampal tissue, and treatment with ghrelin reduces the effect of Aβ. These results are consistent with previous studies on the effect of Aβ and ghrelin on areas of the brain, including the hippocampus, and tissue changes in this area [40,41].

The behavioral assessments in this study showed that ghrelin significantly increased memory function, which supports previous findings on the effect of ghrelin on memory function [22,42,43]. Recent studies showed that ghrelin affects memory and cognition. However, the precise mechanisms underlying ghrelin’s modulation of the memory process are still uncertain [44]. Ghrelin injection into the rat amygdala, hippocampus, and dorsal raphe nucleus increases memory retention [45]. Moreover, ghrelin improves dendritic synapse formation, long-term potentiation (LTP) generation, and spatial learning and memory [43]. Both subcutaneous and intracerebroventricular (i.c.v.) administrations of ghrelin increase learning and memory [43,44]. The ghrelin receptor is present in the hippocampus and the injection of ghrelin agonists enhanced memory retention [46]. In the present study, intrahippocampal injection of Aβ significantly reduced memory function, and ghrelin treatment significantly compensated for it, which is consistent with previous findings regarding the effect of ghrelin on memory in rats with AD [30,41]. We observed that the post-training injection of ghrelin improves the consolidation of hippocampus-dependent forms of memory in the absence of any pathology. Chronic administration of ghrelin may improve memory impairment and alleviate cognitive dysfunction in AD patients [47]. A recent study using nuclear magnetic resonance (NMR) to identify metabolites shows that the intracerebroventricular (i.c.v.) ghrelin injection improves memory and cognitive functions in the animal model of AD [48]. Consistent with these reports, we observed that Aβ-injected rats showed spatial memory deterioration, and ghrelin administrations significantly attenuated this impairment.

Our data show that the hippocampus and serum level of MDA in the ghrelin group was significantly reduced. Numerous studies have been performed to assess the antioxidant effect of ghrelin. The effect of Ghrelin has been reported to reduce the amount of MDA in the brain and plasma of rats. In addition, there is a decrease in antioxidant enzyme activity in the rat plasma [25]. MDA is an end-product of lipid peroxidation. Lipid peroxidation is a critical criterion for oxidative stress and is in the opposite ratio to antioxidant properties. In this study, the hippocampus and serum level of MDA in the Aβ group showed a significant increase. The results support the hypothesis that oxidative stress is involved in AD. Previous studies have shown that Aβ may play a role in mitochondrial damage and increased ROS production. Increased fat peroxidation has been observed in the early stages of AD in patients’ brains [17]. Accordingly, antioxidants, including ghrelin, may be used to treat or prevent AD [19]. Increased lipid peroxidation markers in patients with mild cognitive impairment indicate that increased lipid peroxidation may be a primary event in AD progression [17]. High MDA levels are seen in different brain areas, cerebrospinal fluid (CSF), and serum in patients with AD [18,49]. Moreover, the increase in oxidative stress and peroxidation products results in the overexpression of antioxidant enzymes, such as SOD and heme oxygenase 1 [50,51]. Several hypotheses explain the production of ROS in AD. Some authors believe that the Aβ oligomer potentially produces H_2_O_2_. Others believe that Aβ is involved in the production of ROS by binding to redox-active metal ions or physiological reducing agents such as ascorbate. However, none of these hypotheses completely explain the molecular mechanisms responsible for the source of oxidative stress [52,53]. In the present study, the hippocampal levels of MDA in the group receiving Aβ + ghrelin was significantly decreased compared to the group receiving Aβ. This finding indicates that ghrelin, due to its antioxidant properties, can play a role in reducing the rate of fat peroxidation in AD. To our knowledge, this is the first report showing that ghrelin reduces MDA production in AD. Ghrelin inhibits the activation of microglia inflammation and increases the protein UCP2 in the mitochondria. This protein enhances neuroprotection by reducing the production of ROS and increasing mitochondrial biogenesis. Therefore, the neuroprotective role of ghrelin in the brain may be dependent on UCP2. These neuroprotective effects of ghrelin appear to be mediated by activation of its receptor (GHSR-1) because they are eliminated by drug blocking or genetic deletion of the receptor [54]. Ghrelin was reported to possess anti-oxidative effects in MES23.5 cells that were subjected to the mitochondrial complex 1 inhibitor with 1-methyl-4-phenylpyridinium (MPP), reducing MDA formation in these cells [55,56]. Also, ghrelin has been shown to inhibit the apoptosis-promoting protein glycogen synthase kinase-3, improve Aβ clearance through an unknown pathway and reduce the toxicity of Aβ and inflammatory cytokines in AD [54]. Stimulation of GHSR1a by ghrelin enhances autophagy, prevents the accumulation of Aβ peptides, and facilitates autophagy-mediated degradation of autophagosomes containing Aβ [23]. Numerous studies have been performed on the antioxidant effect of ghrelin. The effect of ghrelin in reducing the amount of MDA in the brain and plasma of rats, and preventing a decrease in antioxidant enzyme activity in the rat plasma has been reported [25]. Ghrelin also promotes antioxidant defense in the liver of the rat [12]. Ghrelin attenuates negative effects on sperm parameters and enhances the fertility of the mice exposed to cyclophosphamide [57]. Antioxidant effects of ghrelin in rat ovaries and testis have been reported [26,27]. Antioxidants have been extensively studied to prevent some diseases. Some natural compounds with antioxidant properties have been studied as adjunctive therapy for some neurological disorders, including AD [58,59]. Such antioxidants have been reported to protect against extracellular and intracellular ROS. Because oxidative stress has been involved in the onset and progression of AD, the possibility of using antioxidants to prevent and treat AD is important. Antioxidant therapy can be considered a promising and low-risk treatment strategy for AD [14,50]. Thus. Ghrelin’s anti-oxidative properties might be beneficial for the treatment of AD.

In the present study, a significant decrease in hippocampus catalase activity was found in the Aβ group, and treatment with ghrelin compensates for this reduction. A decrease in catalase activity has been observed in a streptozotocin-rat model of AD [29]. Previous studies have shown that catalase activity was significantly decreased in the brain of patients with AD [49,60]. Catalase is one of the most critical enzymes in the antioxidant system that converts hydrogen peroxide to water and oxygen [36]. Aβ causes the accumulation of hydrogen peroxide in various ways. It has been observed that direct binding of Aβ to catalase leads to a decrease in catalase activity [61].

In the present study, antioxidant capacity in the hippocampus and serum of rats in the ghrelin receiving group significantly increased. In previous studies, ghrelin prevents a decrease in antioxidant capacity in rat plasma [25]. This change can be attributed to ghrelin’s ability to increase antioxidants and reduce ROS. The serum antioxidant capacity in the Aβ group did not significantly change, but the hippocampal antioxidant capacity reduced, and ghrelin treatment increased this parameter. The low molecular weight antioxidants include ascorbate, uric acid, α-tocopherol, and glutathione, which are important in preventing tissue damage. The FRAP method to evaluate antioxidant capacity is based on reducing Fe^3+^ to Fe^2+^ under acidic conditions. In this situation, hydrophilic materials such as ascorbate are evaluated that can transfer electrons to Fe^3+^ [62]. Ascorbate is a vital antioxidant molecule in the brain that can be rapidly accumulated in the brain and assessed by the FRAP method [63]. Decreased antioxidant activity in CSF and plasma of AD patients can be attributed to a decrease in the amount of ascorbate due to the reaction with ROS in this condition [63,64].

The hippocampus antioxidant capacity level decreased in the Aβ group, and treatment with ghrelin increased it in this study. This change can be due to ghrelin’s effect on reducing ROS. One of the proteins that play an essential role in regulating the antioxidant system is the Nrf2 (nuclear facto-rerythroid 2-related factor 2) protein. Nrf2 can be associated with cellular defense against ROS, and its activity has been observed to decrease with age and with degenerative disorders [65]. Recent studies indicate the role of the Nrf2 signaling pathway in protecting against neurotoxicity induced by Aβ and reducing cell death due to oxidative stress [66]. An association between Aβ, oxidative stress, and Nrf2 can be considered. Recent studies have shown ghrelin’s effect on increasing the expression of the Nrf2 gene and protein in the brain following hemorrhage in mice [67]. Based on this, it can be hypothesized that ghrelin may play a role in strengthening the antioxidant system in the brain in AD by acting on the Nrf2 protein.

In Conclusion, according to the available evidence, lack of protection against ROS production in the brain can be one of the causes of AD progression. Ghrelin can play a protective role in AD due to its antioxidant properties and inhibiting fat peroxidation in the brain.

## Figures and Tables

**Table 1:** The MDA level (nmol/mL), catalase activity (U/mL), and antioxidant capacity (μM) in the rats’ serum in the control, sham, ghrelin, Aβ, and Aβ + ghrelin groups (n = 6).

	Control	Sham	Ghrelin	Aβ	Aβ + ghrelin
MDA level (nmol/mL)	2.1 ± 0.2	2.2 ± 0.1	1.6 ± 0.2*,^a^,^b^,^d^	2.8 ± 0.2*,^a^,^b^,^c^,^e^	1.6 ± 0.2*,^a^,^b^,^d^
Catalase activity (U/mL)	36.8 ± 1.5	34.7 ± 3.0	36.5 ± 4.4	31.7 ± 2.1	35.7 ± 2.1
Antioxidant capacity (μM)	338.3 ± 7.2	330.0 ± 29.1	432.3 ± 42.6*,^a^,^b^,^d^	352.3 ± 16.9*,^c^	407.0 ± 23.6

The data are shown as means ± SD.

* p < 0.05.

a vs control.

b vs sham.

c vs ghrelin.

d vs Aβ.

e vs Aβ + ghrelin.
